# Beyond technology: the dominance of affective factors in explaining speaking ability among Chinese learners using an online platform

**DOI:** 10.3389/fpsyg.2026.1802635

**Published:** 2026-04-29

**Authors:** Wenting Bao, Jinliang Wu, Yadong Zhou, Rujing Zha

**Affiliations:** 1 Department of International Chinese Language Education, School of Humanities and International Education and Exchange, Anhui University of Chinese Medicine, Hefei, Anhui, China; 2 Department of Information Management and Information System, School of Medical Information Engineering, Anhui University of Chinese Medicine, Hefei, Anhui, China; 3 Department of Traditional Chinese Medicine Culture Research, School of Humanities and International Education and Exchange, Anhui University of Chinese Medicine, Hefei, Anhui, China; 4 Department of Psychology, School of Humanities and International Education and Exchange, Anhui University of Chinese Medicine, Hefei, Anhui, China

**Keywords:** affective factors, automatic speech recognition (ASR), Chinese as a second language (CSL), online learning, speaking proficiency

## Abstract

**Introduction:**

As the number of international students learning Chinese as a foreign language continues to rise, Chinese language teaching and learning face growing challenges. This study explores and compares the relative explanatory contribution of affective and platform-perception variables to Chinese speaking proficiency among learners using an online learning platform.

**Methods:**

A total of 113 beginner-level CSL learners participated in a short GCLP-supported classroom intervention. Learners completed measures of willingness to communicate, speaking anxiety, motivation to speak, self-esteem in speaking, perceived usefulness, and perceived ease of use. Correlation analyses and two-block hierarchical regression were conducted to examine relationships with Chinese speaking performance and to compare the explanatory contribution of affective versus platform-perception variables.

**Results:**

Willingness to communicate, motivation to speak, and self-esteem in speaking were positively associated with Chinese speaking performance, whereas speaking anxiety was negatively associated. Regarding platform-perception variables, perceived usefulness of the technology used for speaking practice and feedback was positively associated with speaking ability, whereas perceived ease of use of that technology was not significantly associated with speaking proficiency. Hierarchical regression further showed that affective variables accounted for substantially more variance in Chinese speaking performance than learners’ perceptions of the technology used in this study.

**Discussion:**

These findings suggest that, in this short ASR-supported CSL speaking context, affective variables are more relevant than baseline platform perceptions in explaining performance differences. The results highlight the importance of affect-supportive pedagogy and platform design in technology-mediated Chinese speaking instruction.

## 1 Introduction

With China’s rapid economic growth and expanding global market opportunities, an increasing number of international students are pursuing Chinese as a second language (L2). This trend, however, has created challenges regarding the shortage of qualified teachers and sufficient educational resources (Gong et al., 2020). Since the reform and opening up policy initiated in the late 1970s, China has experienced substantial growth in foreign exchanges, significantly boosting interest in Chinese language teaching and learning (Hu and Guo, 2021). Particularly, China’s strategic initiative, the “Belt and Road” policy, which encompasses 65 countries and approximately 50 languages, has significantly amplified enthusiasm for Chinese language acquisition among learners from diverse linguistic and cultural backgrounds, presenting both opportunities and challenges for Chinese language teaching and learning (Feng et al., 2020).

To address this issue, many scholars and educators have turned to technological resources to enhance educational accessibility (Ruan and Medwell, 2019; Yu, 2022; Valverde-Berrocoso et al., 2021). Despite these technological advancements, L2 speaking outcomes remain unsatisfactory, with learners often demonstrating passivity during speaking tasks (Marcellino, 2005). The effectiveness of Chinese language teaching and learning continues to be compromised by various limitations, including insufficient personalized instruction to support affective dereference in online environment (Cobos, 2023; Qi et al., 2024) and a lack of suitable course designs of integrating technology into language classes (Golonka et al., 2014; Kukulska-Hulme, 2013).

Previous research indicates that both affective factors (Afifah et al., 2024) and technology-adoption variables (Alfadda and Mahdi, 2021) shape learners’ oral performance. Although digital tools are now indispensable, a growing body of work shows that learners’ affective readiness, for example, willingness to communicate (WTC), motivation, anxiety, and self-esteem, plays a crucial role in technology-mediated learning, especially for productive skills such as speaking (Bielak and Mystkowska-Wiertelak, 2024; Teimouri et al., 2019). Many studies (Saito and Akiyama, 2017; Stander, 2022), however, have examined technology acceptance and affective factors in isolation, leaving unanswered the practical question of how these factors influence language learning and which set of variables matters more when the two are modelled simultaneously.

This gap is particularly salient for Chinese as a L2 (CSL) contexts, where the empirical base remains heavily skewed toward English L2 settings (ESL) (Wu et al., 2025). Chinese language differs fundamentally from English: it is a lexical-tone language written with logographic characters. Consequently, learners must master two intertwined phonological layers, involving accurate tone perception and the precise mapping of tones onto individual characters—requirements that go well beyond the segmental, alphabetic focus of typical EFL classrooms. Empirical evidence illustrates these differences; for instance, in synchronous computer-mediated classes, the efficacy of corrective feedback diverges between tone and non-tone languages (Bryfonski and Ma, 2020), and the benefits of acoustic exaggeration during perception training vary by tone category, underscoring the need for tone-specific adaptive algorithms (Cao et al., 2024). Recent automatic speech recognition (ASR) research further suggests that character-level feedback is especially beneficial, because each character carries its own tone slot, enabling fine-grained diagnosis of pronunciation errors (Li et al., 2023).

Identifying and weighing the affective and technological determinants of CSL speaking performance is therefore essential for optimising instruction. The present study addresses this need by comparing the relative influence of affective and technological variables on learners’ Chinese speaking proficiency within the Global Chinese Learning Platform (GCLP), an ASR-enabled platform that provides character-level pronunciation feedback. Findings are intended to inform both course design and platform development in Chinese language education. The contribution of the present study lies less in identifying wholly novel correlations than in extending this line of inquiry to a multilingual CSL context and directly comparing the relative explanatory contribution of affective versus platform-perception variables within the same ASR-supported classroom setting. More specifically, the study contributes by showing that, in this short ASR-supported CSL context, affective variables were more relevant than platform-perception variables in accounting for differences in speaking performance.

## 2 Theoretical background and conceptual framework

### 2.1 Affective factors

Krashen’s Affective Filter Hypothesis and MacIntyre’s Willingness to Communicate model jointly explain why learners’ emotions and dispositions shape their oral outcomes. Krashen (1982) posits that motivation, self-confidence, and anxiety modulate a mental “filter” between input and intake. Large scale surveys and classroom experiments consistently confirm these links. For example, it is reported that motivation and self-esteem together explained around one-third of the variance in university English learners’ end-of-term oral scores (Bao and Liu, 2021), while it is also showed that positive attitudes toward English accounted for roughly three-tenths of the variance in self-reported speaking ability among a sample of Ethiopian tenth-graders (Getie, 2020). Anxiety pulls in the opposite direction: in a longitudinal study of Chinese postgraduate students, speaking anxiety correlated negatively with oral-proficiency scores, showing that highly anxious learners gained considerably less improvement in oral proficiency than their low-anxiety peers (Woodrow, 2006).

Complementing Krashen, MacIntyre conceptualises WTC as “the probability that a learner will initiate communication when given the choice” (McCroskey and Richmond, 1990). We also acknowledge that WTC can be conceptualized both as a relatively stable disposition and as a situationally fluctuating state; the present study focuses on a generalized self-reported tendency rather than moment-to-moment variation. Its predictive power has repeatedly surpassed that of formal aptitude tests: in a Turkish preparatory-class sample, WTC correlated with instructor-rated speaking proficiency (Bergil, 2016). Recent Chinese language research echoes this pattern: it is found that WTC and L2 grit jointly predicted young heritage learners’ picture-description scores (Sun et al., 2024). Taken together, these findings justify our focus on four affective factors, including WTC, anxiety to speak, motivation to speak, and self-esteem in speaking, as direct correlates of Chinese speaking performance.

### 2.2 Technological factors

In the present study, technological factors refer to learners’ perceptions of the GCLP as an external instructional tool rather than to stable trait-like learner characteristics. Specifically, we focus on perceived usefulness (PU) and perceived ease of use (PEOU), two core belief constructs from Davis’s (1989) Technology Acceptance Model. Examining these perceptions can help clarify how learners experience a digital speaking environment, even when the present design does not allow direct conclusions about the technical effectiveness of the platform itself. Recent work illustrates how PU and PEOU operate in real classrooms. One study of Saudi ESL learners showed that PU and PEOU jointly explained a substantial proportion of learners’ performance in using Zoom during emergency remote teaching (Pan et al., 2023). Another large-scale survey of mobile learning in Indian higher education showed that both PU and PEOU were associated with self-reported academic achievement and related learning beliefs (Panigrahi et al., 2021).

### 2.3 Present study

**Figure 1:**
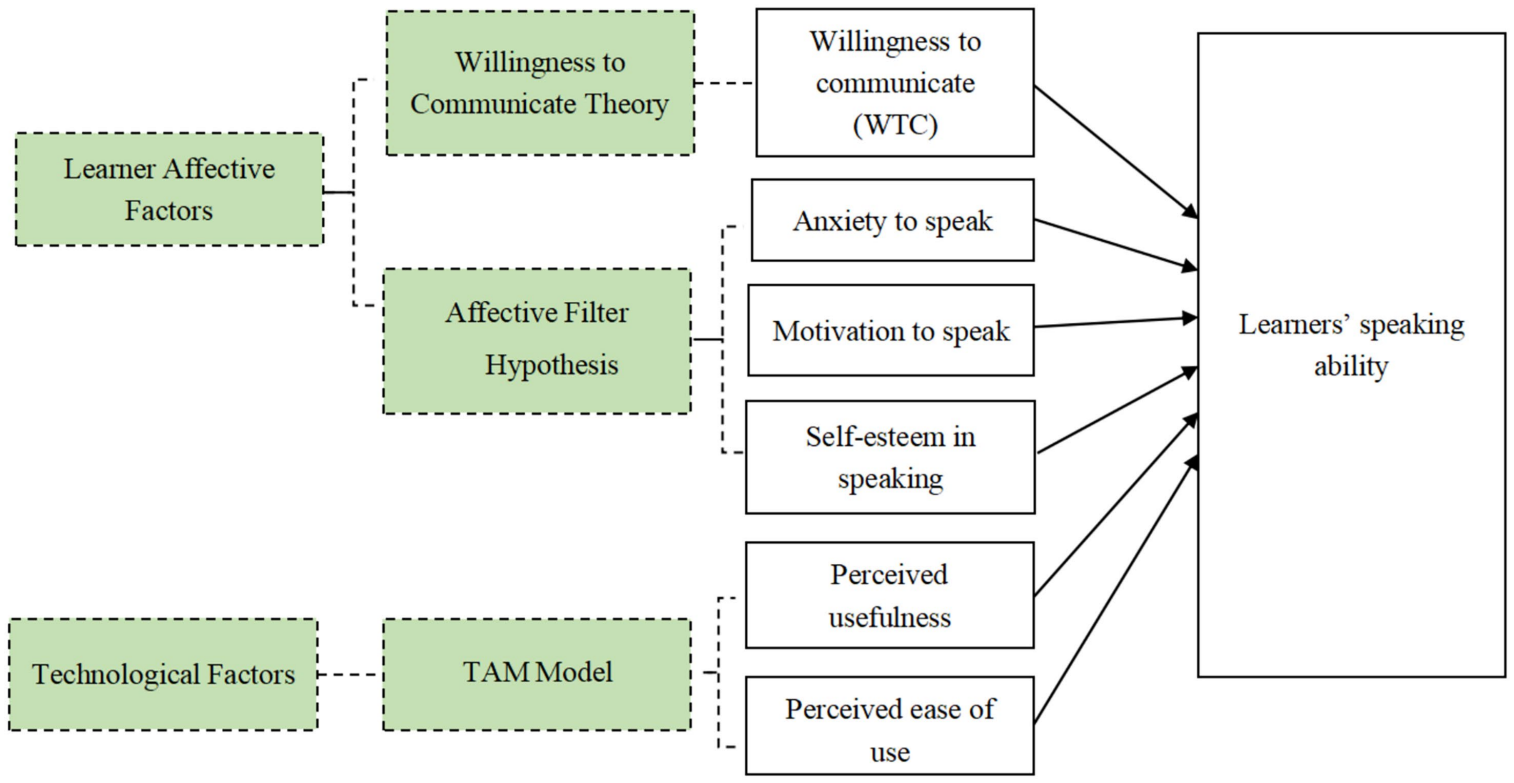
Conceptual framework of this study.

Although online learning research frequently adopts technology models such as the Technology Acceptance Model to examine learners’ technology perceptions or focuses on affective constructs grounded in Krashen’s affective filter and the WTC tradition, these strands are typically examined in isolation (King and He, 2006; Peng et al., 2023; Wang et al., 2025). Few studies have subjected them to a parallel comparison within the same statistical model. Building on recent integrative work, the present study does not attempt to test a full causal technology-adoption sequence. Instead, it compares two conceptually distinct but contextually relevant sets of variables within the same ASR-supported classroom setting: (a) affective learner variables and (b) learners’ perceptions of the external platform. Because the intervention lasted four 40-min classroom sessions and all tasks were completed under teacher-organised conditions, actual system use showed no between-learner variance and behavioural intention could not be meaningfully distinguished from compliance. We therefore adopted a two-block hierarchical regression approach to examine which set of variables accounted for more variance in speaking performance in this specific instructional context. Consistent with this design, PU and PEOU are analysed here as post-exposure platform perceptions alongside affective variables, rather than as evidence of the platform’s effectiveness per se (see Figure 1).

Specifically, this study is guided by the following research questions (RQs):

- RQ1: What are the relationships between learner affective factors (i.e., willingness to communicate, anxiety to speak, motivation to speak, and self-esteem in speaking) and learners’ Chinese speaking ability?
- RQ2: How are learners’ perceptions of the GCLP (i.e., perceived usefulness and perceived ease of use) associated with their Chinese speaking performance?
- RQ3: In this short ASR-supported classroom context, which set of variables—affective factors or platform-perception variables—accounts for more variance in Chinese speaking performance when modelled together?

## 3 Methodology

### 3.1 Data collection

This study is part of a larger research project investigating the effects of affective and technological factors on L2 learners’ Chinese speaking abilities. Ethical approval was obtained from the Universiti Malaya Research Ethics Committee (UMREC, Non-Medical) (Approval No. UM.TNC2/UMREC_4566), valid from May 2025 to May 2028. Permission to conduct the study was granted by the participating universities, with class instructors facilitating access to students.

A purposive sampling method was applied because the study required participants with a specific and relatively homogeneous instructional profile: learners who had studied Chinese for at least one semester, had not yet passed HSKK Level 3, and were enrolled in the same HSKK3 preparation programme in which the GCLP-supported speaking tasks could be embedded. Participation was voluntary, anonymous, and confidential. Participation was organised on an opt-in basis in May 2025: only students who voluntarily joined the GCLP speaking project were invited to complete the research instruments, and they were informed that they could withdraw at any time without penalty. Because the tasks and questionnaires were administered during class time to this consenting group, completion was 100%. Questionnaires were returned directly to the research team; the optional student-ID field was used only for potential follow-up and was removed before analysis to preserve confidentiality.

All participants were fully informed about the study’s objectives, procedures, confidentiality, and their rights, including the voluntary nature of participation and the option to withdraw at any time without any consequences. Written informed consent was obtained on-site. Only university-level students aged 18 years or older were recruited; therefore, parental or guardian consent was not required.

### 3.2 Experiment

This exploratory quasi-experiment was embedded in the regular curriculum. Over 2 weeks, students attended four 40-min speaking classes and completed four classroom-based dialogue tasks using the ASR tutor on the GCLP online platform. The tasks focused on familiar HSKK3-related themes, such as self-introduction, daily routines, campus life, and simple interpersonal exchanges. In each task, learners first viewed the prompt and then produced spoken responses through the GCLP interface. The ASR system analysed each spoken syllable against the target character and returned colour-coded feedback indicating whether pronunciation was accurate, partially accurate, or in need of repetition. All speaking tasks were completed in class, and platform logs confirmed 100% task completion, yielding no meaningful between-subject variance in actual usage. Immediately after the final session, students completed a questionnaire capturing their post-exposure affective and platform-perception responses.

### 3.3 Instruments

Since the cohort was multilingual and no single L1 could cover all participants, the instruments were prepared in parallel English and Chinese versions. Participants completed the English version, with brief clarificatory notes provided where needed, to ensure that item meanings were not lost for learners from different backgrounds. Six instruments were employed in the study: (1) the HSKK test and five questionnaires, (2) the Willingness to Communicate Questionnaire (MacIntyre et al., 2001), (3) the Foreign Language Speaking Anxiety Scale (Ozturk and Gurbuz, 2014), (4) the Attitude and Motivation Test Battery (Gardner, 1985), (5) the Foreign Language Self-Esteem Scale (Hassan, 1992), and (6) the Technology Acceptance Model scale measuring PU and PEOU (Davis, 1989). All five questionnaires were subsequently adapted and re-validated for the multilingual Chinese-learning context following the procedures detailed in previous published research related to this study (Bao and Yusop, 2025).

Each draft underwent back-translation procedure to produce bilingual (Chinese and English) versions to get experts’ opinions (Wang et al., 2021). An 11-member panel from domestic and overseas universities rated item essentiality. The Content Validity Ratio (CVR) and Content Validity Index (CVI) were computed following established procedures (Alqahtani et al., 2023). Any item that more than half of the expert panel deemed “not essential,” or that would have lowered the scale-level CVI below 0.70, was removed to optimize content validity; the resulting instruments achieved final CVIs ranging from 0.71 to 0.84. The revised questionnaires were piloted with 54 first-year Chinese-language learners whose profile matched the target sample. Internal consistency analysis produced satisfactory Cronbach’s *α* coefficients for every construct (WTC = 0.83, Anxiety = 0.96, Motivation = 0.89, Self-esteem = 0.92, PU = 0.96, and PEOU = 0.94), exceeding the 0.80 benchmark for research use. These sequential procedures establish strong content validity and reliability, supporting the instruments’ suitability for the main study. The final retained items were: WTC 7 items, Speaking-Anxiety 16 items, Motivation 17 items, Speaking-Self-Esteem 16 items, PU 6 items, and PEOU 6 items, with a total number of 68 items.

#### 3.3.1 HSKK test

The Hanyu Shuiping Kouyu Kaoshi (HSKK) is the oral branch of the International Chinese Proficiency Test and offers standardized, level-linked tasks for assessing speaking proficiency (Teng, 2017). Generalizability analyses report excellent reliability (Ep^2^ ≈ 0.93) and strong criterion validity across item types (Zhao, 2006). In the present study, past official HSKK-3 papers were embedded in the GCLP online platform as mock tests to gauge learners’ Chinese-speaking ability. All responses were scored by two trained raters using the official analytic rubric; inter-rater reliability was high (ICC = 0.92, 95% CI [0.89, 0.95]), confirming the robustness of the proficiency measure. Therefore, students’ Chinese speaking ability is as indexed by performance on standardised, level-linked oral tasks and measured by HSKK mock test in this study.

#### 3.3.2 Willingness to communicate (WTC) questionnaire

Adapted from MacIntyre et al. (2001), this 16-item instrument measures the affective construct WTC, which is learners’ readiness to initiate spoken interaction inside and outside class. Because the present intervention targeted in-class, ASR-supported Chinese speaking, items referring to out-of-class or unspecified L2 communication were removed to keep the construct aligned with the classroom opportunity structure; the adapted version retained good internal consistency (*α* = 0.83). Therefore, WTC Questionnaire is applied to measure students’ WTC in Chinese language in this study. All retained WTC items were rated on a five-point Likert scale ranging from 1 (strongly disagree) to 5 (strongly agree).

#### 3.3.3 Foreign language speaking anxiety scale

Derived from the speaking subset of the Foreign Language Classroom Anxiety Scale (Horwitz et al., 1986) and refined by Ozturk and Gurbuz (2014), the 18-item scale gauges anxiety to speak by asking respondents to indicate their apprehension during oral tasks on a five-point Likert scale from “strongly disagree” to “strongly agree.” Minimal localization was conducted to ensure cultural relevance while preserving the parent scale’s high reliability. Therefore, anxiety to speak Chinese refers to the apprehension or tension experienced during L2 oral tasks and it is measured by Foreign Language Speaking Anxiety Scale in this study.

#### 3.3.4 Attitude and motivation test battery

Gardner’s (1985) Attitude and Motivation Test Battery questionnaire was condensed to 20 items covering interest, motivational intensity, integrative orientations, and classroom attitudes, thereby used to measure motivation to speak in this study. All items were contextualized for the current setting and scored on a five-point Likert scale to align with the other instruments. Previous studies confirm the reliability of the Attitude and Motivation Test Battery questionnaire, with cross-cultural research consistently reporting strong psychometric properties (Shinge and Kotabagi, 2021). Therefore, motivation to speak refers to the effort, interest, and orientation toward engaging in L2 oral practice and it is measured by Attitude and Motivation Test Battery questionnaire in this study.

#### 3.3.5 Foreign language self-esteem scale

Hassan’s (1992) inventory was streamlined to 16 statements assessing learners’ evaluative self-beliefs in oral communication, which is self-esteem in speaking in this study. Content localization and proficiency-level adjustments were applied, and responses were captured on a five-point Likert scale from “strongly disagree” to “strongly agree.” Previous research shows excellent internal consistency (*α* = 0.95) and inter-rater agreement (*r* = 0.81) of this questionnaire. Therefore, self-esteem in speaking refers to the self-evaluation and confidence in one’s ability to communicate orally in the L2, and it is measured by Foreign Language Self-Esteem Scale in this study.

#### 3.3.6 Technology acceptance model questionnaire

Based on Davis (1989), this 12-item tool measures two platform-perception variables, which are PU and PEOU, with six items per factor. Statements were localized to the study’s speaking application in GCLP, and all items were rated on a five-point Likert scale ranging from 1 (strongly disagree) to 5 (strongly agree). Technology Acceptance Model measures routinely exhibit strong convergent and discriminant validity in educational technology contexts (Wu and Wang, 2005). In the present study, PU refers to the degree to which GCLP is perceived as helpful for speaking practice, whereas PEOU refers to the degree to which using the GCLP online platform is perceived as easy and manageable. The Technology Acceptance Model questionnaire was used to measure students’ PU and PEOU in this study.

### 3.4 Participants

A total of 121 beginner-level Chinese language learners preparing for HSKK3 completed all measures. Inclusion criteria were: (i) studied Chinese for at least one semester, (ii) had not passed HSKK-3, and (iii) had been placed by the university in the same HSKK3 preparation programme on the basis of comparable Chinese test scores. There were no missing data or format errors.

For data screening, score-based outliers were identified with Tukey’s 1.5 × IQR rule from boxplots (5 cases removed). In addition, because the questionnaire contained 68 Likert-type items covering six distinct constructs, response patterns that selected the same option across all items were treated as invalid extreme-response style and excluded (3 cases, 2.7%) (Van Vaerenbergh and Thomas, 2013). After deleting the extreme data, the final sample comprised of 113 international students from one university in Anhui Province, China. This exceeds the common rule of thumb for multiple regression (≥10 cases per predictor) (Schreiber et al., 2006). An a-priori G*Power analysis for linear multiple regression (6 predictors, two-tailed, *α* = 0.05, power = 0.80) targeting f^2^ = 0.10 recommended at least 98 participants; our sample therefore meets this criterion. After data collection, a post-hoc power check for the full regression model based on the observed *R*^2^ = 0.592 indicated power > 0.99.

These 113 participants included 60 males and 53 females, ranging in age from 18 to 35 years old (M = 23, SD = 3.87). Students came from 34 countries across Africa, Asia, North America, and Europe, providing substantial multilingual and multicultural diversity (Table 1).

**Table 1:** Students’ countries and L1s.

Continent	Country	First language	Numbers
Africa	Algeria	Arabic, Berber	8
	Benin	French	1
	Botswana	Tswana	2
	Burkina Faso	French	1
	Togo	French, Ewe	2
	Democratic Republic of the Congo	French	1
	Ghana	English, Akan	4
	Zimbabwe	English, Shona	3
	Cameroon	French, English	4
	Ivory Coast	French	1
	Kenya	English, Swahili	1
	Liberia	English, Kpelle	2
	Rwanda	Kinyarwanda, French, English	4
	Morocco	Arabic, Berber	14
	Nigeria	English, Hausa	3
	South Africa	English	1
	Sudan	Arabic, English	10
	Somalia	Somali	1
	Tanzania	Swahili, English	5
	Uganda	English, Luganda	2
	Zambia	English, Bemba	3
	Chad	French, Arabic	1
Asia	Afghanistan	Pashto	1
	Pakistan	Urdu	6
	Kazakhstan	Kazakh	4
	South Korea	Korean	5
	Malaysia	Malay	10
	Thailand	Thai	2
	Vietnam	Vietnamese	3
Europe	Russia	Russian	4
	Ireland	Irish	1
North America	USA	English	1
	Canada	English	1
Total number of participants			113

### 3.5 Data analysis

The post-intervention HSKK score was used as the speaking-performance outcome in all analyses. All inferential tests were two-tailed with *α* = 0.05. For each variable we inspected histograms and Q-Q plots, computed skewness/kurtosis (|skew| < 3, |kurtosis| < 7 as acceptable), and ran Shapiro–Wilk tests (plus Kolmogorov–Smirnov for HSKK). Variables meeting normality were analysed with Pearson’s *r*; otherwise with Spearman’s *ρ*. We report effect sizes with 95% CIs (Fisher’s z for *r*; bias-corrected and accelerated [BCa] bootstrap with 5,000 resamples for *ρ*).

For scale construction, reverse-keyed items were recoded and subscales were computed as item-mean composites to retain equal weights and interpretability. Internal consistency was re-estimated on the study sample (Cronbach’s *α* and McDonald’s *ω*; 0.83 ≤ *α* ≤ 0.96). The six composites (WTC, anxiety, motivation, self-esteem, PU, PEOU) were z-standardised prior to correlations and regression.

To compare affective versus technological contributions, we fitted a two-block hierarchical linear regression (Block 1: affective; Block 2: technology) in SPSS 26 and examined ΔR^2^. Diagnostics included multicollinearity (VIF < 2.5; tolerance > 0.40), residual normality (Shapiro–Wilk), and linearity/homoscedasticity (residual plots; Breusch-Pagan). Given mild heteroscedasticity, we used HC3 heteroskedasticity-consistent standard errors for inference (Davidson and MacKinnon, 1993).

## 4 Results

### 4.1 Descriptive statistics and normality test

Table 2 summarises descriptive statistics for the HSKK test and the six questionnaire scores. Also, normality diagnostics showed that the HSKK scores, along with the WTC and anxiety composites, were normally distributed and were therefore analysed with parametric tests (Pearson *r*). By contrast, motivation, self-esteem, PU, and PEOU all departed from normality and were treated with non-parametric statistics (Spearman *ρ*).

**Table 2:** Descriptive statistics.

Factor	*N*	Mean	SD
HSKK pre-test	113	46.97	12.20
HSKK post-test	113	51.54	12.33
Willingness to communicate (WTC)	113	2.78	0.83
Speaking anxiety	113	3.01	0.86
Motivation to speak	113	3.12	0.78
Speaking self-esteem	113	3.04	0.77
Perceived usefulness (PU)	113	3.19	0.67
Perceived ease of use (PEOU)	113	3.51	0.60

HSKK scores ranged from 21 to 77 in the present sample.

### 4.2 Relationships between affective factors and speaking ability

To clarify how affective dispositions shape oral performance, four learner affective variables—WTC, anxiety, motivation, and self-esteem—were illustrated to be correlated with HSKK scores:

#### 4.2.1 Willingness to communicate

WTC correlated strongly and positively with speaking ability (*r* = 0.746, *p* < 0.001), accounting for more than half of the shared variance. Learners who reported a greater readiness to initiate conversation consistently achieved higher HSKK scores.

#### 4.2.2 Speaking anxiety

In contrast, anxiety displayed a moderate, negative association (*r* = −0.429, *p* < 0.001). Elevated nervousness and tension during oral tasks thus coincided with noticeably lower performance.

#### 4.2.3 Motivation to speak

Motivation showed a moderate, positive relationship with speaking outcomes (*ρ* = 0.453, *p* < 0.001). Students who valued and enjoyed practising oral Chinese tended to perform better, although the effect was smaller than that of WTC.

#### 4.2.4 Speaking self-esteem

A similar moderate, positive correlation emerged for self-esteem (*ρ* = 0.457, *p* < 0.001). Learners with higher self-evaluations of their speaking competence demonstrated superior HSKK scores.

Taken together, the pattern is consistent with affective-filter theory: facilitative emotions such as high WTC, motivation, and self-esteem coincide with better speaking proficiency, whereas higher speaking anxiety is associated with lower speaking performance.

### 4.3 Relationships between technological factors and speaking ability

This section examines whether learners’ technology-related beliefs—PU and PEOU—bear on their Chinese speaking performance.

#### 4.3.1 Perceived usefulness (PU)

PU exhibited a weak yet statistically reliable positive correlation with HSKK scores (*ρ* = 0.345, *p* < 0.001). Learners who regarded the ASR-based platform as helpful for improving their speaking tended to achieve modestly higher test results.

#### 4.3.2 Perceived ease of use (PEOU)

In contrast, PEOU was not significantly related to speaking ability (*ρ* = 0.151, *p* = 0.109). No statistically significant association was observed between PEOU and speaking performance in this sample.

To holistically illustrate the effects of affective (RQ1) and technological (RQ2) factors on Chinese speaking ability, Table 3 consolidates correlation coefficients (*r/ρ*), 95% confidence intervals (CIs), and significance levels across all six variables.

**Table 3:** Synthesized analysis of RQ1 and RQ2.

Factor	Type	r/*ρ*	95% CI	*p*	Effect size
WTC	Pearson *r*	0.746	[0.654, 0.815]	<0.001	Large
Anxiety	Pearson *r*	−0.429	[−0.550, −0.290]	<0.001	Moderate
Motivation	Spearman *ρ*	0.453	[0.280, 0.595]	<0.001	Moderate
Self-Esteem	Spearman *ρ*	0.457	[0.285, 0.599]	<0.001	Moderate
PU	Spearman *ρ*	0.345	[0.170, 0.495]	<0.001	Weak
PEOU	Spearman *ρ*	0.151	[−0.030, 0.325]	0.109	n.s.

### 4.4 Comparison between affective and technological factors

Table 4 presents the means, standard deviations, reliability coefficients, and zero-order correlations among the HSKK pre−/post-test scores and all affective and technology variables.

**Table 4:** Intercorrelations among study variables.

Variable	M	SD	α	1	2	3	4	5	6	7	8
1. HSKK post-test	51.54	12.33	—	—							
2. HSKK pre-test	46.97	12.20	—	0.98**	—						
3. WTC	2.78	0.83	0.83	0.75**	0.73**	—					
4. Anxiety	3.01	0.86	0.96	−0.43**	−0.43**	−0.37**	—				
5. Motivation	3.12	0.78	0.89	0.34**	0.33**	0.43**	−0.35**	—			
6. Self-esteem	3.05	0.77	0.92	0.41**	0.40**	0.48**	−0.19*	0.34**	—		
7. PU	3.19	0.67	0.96	0.33**	0.34**	0.44**	−0.13	0.32**	0.51**	—	
8. PEOU	3.51	0.60	0.94	0.17	0.18	0.15	0.02	−0.03	0.24*	0.27**	—

*p < 0.05; **p < 0.01.

To address RQ3, whether affective or technological variables account for greater variance in Chinese speaking ability, two sets of hierarchical regression analyses were conducted using HC3 heteroscedasticity-robust standard errors. Four models were tested to evaluate the incremental contribution of each variable group (Table 5).

**Table 5:** Hierarchical regression.

Model	Variables included	*R* ^2^	Δ*R*^2^	F-change	*p*
1	Affective	0.587	—	—	—
2	Affective + Technological	0.592	+0.004	0.53	0.593
3	Technological	0.118	—	—	—
4	Technological + Affective	0.592	+0.473	30.69	<0.001

Δ*R*^2^ represents the incremental variance explained compared to the baseline model (e.g., Model 2 vs. Model 1; Model 4 vs. Model 3).

When affective factors were entered first (Model 1), they explained 58.7% of the variance in speaking performance. Adding technological factors in Model 2 resulted in only a minimal and non-significant increase (ΔR^2^ = 0.004, F-change = 0.53, *p* = 0.593), suggesting that technological variables provided little additional explanatory power beyond affective factors. In contrast, when technological factors were entered first (Model 3), they accounted for only 11.8% of the variance. However, introducing affective factors in Model 4 significantly improved the model’s explanatory power by an additional 47.3 percentage points (ΔR^2^ = 0.473, F-change = 30.69, *p* < 0.001), indicating that affective variables play a much more substantial role in Chinese speaking ability.

Robustness checks confirmed the reliability of these findings. No evidence of multicollinearity was detected (all VIFs ≤ 2.5), residuals approximated normality (Shapiro–Wilk *W* = 0.984, *p* = 0.216), and the mild heteroscedasticity flagged by the Breusch-Pagan test (*p* = 0.017) was handled with HC3 heteroskedasticity-consistent standard errors. The HC3 estimator, proposed by Davidson and MacKinnon (1993), applies an adjustment to each squared residual and, compared with the more commonly used HC0–HC2 variants, provides a stronger small-sample bias correction for *t* statistics.

In summary, the results clearly demonstrate the stronger explainable value of affective factors compared to technological factors. The technological factors offered almost no meaningful additional contribution once affective variables were considered. These findings highlight the importance of psychological and affective dimensions in shaping Chinese speaking outcomes in technology-assisted language learning environment.

## 5 Discussion

### 5.1 Interpretation of core findings

The findings of RQ1 illustrate the crucial role of affective factors in CSL speaking classes. Specifically, all of the four learner affective variables, which are WTC, anxiety to speak, motivation to speak, and self-esteem in speaking, showed a significant correlation with Chinese speaking ability. This result aligns with many studies in L2 acquisition emphasizing that learners’ affective states critically shape learners’ communication outcomes. For example, WTC is found to be highly correlated with language learners’ speaking proficiency, suggesting that learners with higher WTC may be more inclined to engage in speaking opportunities, which is consistent with stronger oral performance (Peng and Woodrow, 2010; Sato, 2020). In contrast, language anxiety has long been recognized as a major “barrier” to language acquisition, with higher anxiety linked to poorer language performance (Matsuda and Gobel, 2004). Moreover, motivation was illustrated to be positively related to language learning outcomes, as students with high motivation may be more likely to engage actively in language practice, which is consistent with stronger language performance (Pranawengtias, 2022). Self-esteem is also closely found to be related to oral proficiency in language learning, since learners with a growth mindset usually exhibit higher levels of self-esteem in language learning, which makes them more confident in their oral interactions (Ozdemir and Papi, 2021; Inagaki, 2022).

These results are broadly consistent with Krashen’s Affective Filter Hypothesis and suggest that affective readiness is closely related to oral performance in this Chinese online learning context. This study provided an empirical support for understanding Affective Filter Hypothesis by showing how affective factors are closely associated with language performance in a real Chinese online learning context. Unlike the majority of L2 investigations that centre on English, the present study focuses on international students learning Chinese in a tonal, character-based environment—a setting that imposes substantially greater linguistic and psychological demands. Whereas English ASR systems typically supply only utterance-level intelligibility scores, the GCLP returns a colour-coded accuracy rating for each individual character learners pronounce. Because every written character corresponds to a tone-bearing monosyllable, this per-character feedback pinpoints segment-plus-tone errors with high precision, offering a form of pronunciation guidance unavailable in English-oriented ASR tools (Li et al., 2023). These technological and linguistic specificities indicate that findings derived from English-based ASR research cannot be transferred uncritically to Chinese. Future CSL platforms must therefore integrate tone-sensitive feedback mechanisms alongside robust affective-support features if they are to meet the distinctive requirements of CSL learning. This study not only enriches the understanding of Affective Filter Hypothesis theory, but also provides new perspectives and empirical evidence for the study of CSL.

The GCLP used in this study was not selected as a neutral technological background only. Its design features make it theoretically relevant to affective variables as well. Specifically, the platform provides specific, immediate, character-level feedback without direct interpersonal judgement, which may help reduce evaluative pressure during speaking practice. In addition, because learners can rehearse orally in a relatively private and low-stakes environment, the platform may plausibly support willingness to communicate by making oral production feel safer and more manageable. At the same time, its structured feedback may help learners monitor progress and develop more positive self-evaluative speaking experiences. These possibilities are broadly consistent with earlier work suggesting that technology-mediated speaking environments can shape learners’ affective readiness (Cobos, 2023; Qi et al., 2024; Bielak and Mystkowska-Wiertelak, 2024; Teimouri et al., 2019). However, the present findings further indicate that, even in a platform environment likely to support such affective processes, learners’ affective variables themselves were more relevant than their perceptions of the platform in explaining differences in speaking performance.

The results for RQ2 suggest that platform-perception variables were less strongly related to speaking performance than affective variables in this sample. PU showed a weak but significant positive association with HSKK scores, whereas no statistically significant association was observed for PEOU. These findings should be interpreted cautiously. First, the present design does not establish whether more positive platform perceptions lead to stronger speaking performance, or whether stronger speakers are more likely to evaluate the platform more positively. Second, because the intervention was brief and classroom-embedded, PEOU may have had limited opportunity to differentiate learners meaningfully. In such a context, platform usability may function more as a background enabling condition than as a direct correlate of performance differences. The present results therefore do not invalidate the Technology Acceptance Model; rather, they suggest that in short, mandatory ASR-supported CSL practice, baseline ease-of-use perceptions may be less salient than affective readiness in accounting for differences in speaking outcomes. In addition, because participants likely varied in their prior experience with digital learning tools, their ease-of-use ratings may have clustered within a relatively narrow range. This restricted variability, rather than the absence of usability relevance, may have contributed to the non-significant PEOU–HSKK association.

In addition, the hierarchical regression analysis of RQ3 provides compelling evidence that affective variables accounted for substantially more variance than platform-perception variables in this short ASR-supported classroom context. When affective variables were entered first, they accounted for 58.7% of variance in Chinese speaking ability, which is a very large effect. Adding technological factors in the next step contributed almost no additional explanatory power (ΔR^2^ = 0.004). This means that after considering learners’ affective factors, knowing their opinions about the platform’s usefulness or ease added almost nothing to predicting how well they speak in Chinese. The second model strengthened the unique role of affective factors. When entering technology variables alone first explained only about 11.8% of variance, and the subsequent inclusion of affective factors led to a huge jump of 47.3% in R^2^, a significant increase. The contrast between these two models reinforces that it is the learners’ affective readiness and desire to communicate, rather than baseline platform perceptions, affective readiness appeared to account for more of the observed performance variation in this sample. This finding extends prior work on learner psychology by showing that, in this short GCLP-supported CSL speaking context, affective variables accounted for substantially more variance in speaking performance than learners’ perceptions of the platform. This pattern is consistent with the task and feedback design of the platform: colour-coded, character-level feedback makes pronunciation and tone errors highly visible and invites immediate repetition, so learners’ willingness, their motivation, and their self-evaluation become more decisive than their general belief that the tool is easy to use. At the same time, we do not claim that technology beliefs are unimportant in all CSL e-learning settings; rather, our results indicate that under short, mandatory ASR practice with very salient feedback, affective resources can displace PU/PEOU in explaining performance differences. This finding extends prior research on learner psychology by quantifying the circumstances under which affective factors can surpass technology-related factors for complex outputs such as speaking ability, and it suggests that technology may be more pedagogically meaningful when it supports WTC, motivation, and anxiety reduction in ways that help learners engage more confidently in oral practice.

### 5.2 Implications for language teaching and learning

These findings provide important implications for language educators and developers of technology-assisted language learning. As noted in the Introduction, online Chinese learning environments often face insufficient personalised support for learners’ affective needs (Cobos, 2023; Qi et al., 2024), and technology integration is not equally effective when affective dimensions are overlooked (Golonka et al., 2014; Kukulska-Hulme, 2013). The present findings add to this line of reasoning by showing that, even when platform perceptions are considered, affective variables remain more relevant than baseline platform perceptions in explaining speaking performance differences.

On the one hand, when teachers integrate technology, affective support should be placed at the core of course design. The GCLP used in this study already appears to offer some affect-relevant affordances: it provides specific feedback without direct interpersonal judgement, allows relatively private oral rehearsal, and gives learners a structured space for repeated speaking practice. These features may help reduce speaking pressure and make oral participation feel more manageable. At the same time, teachers may further strengthen such platforms by integrating complementary tools or practices that more directly foster willingness to communicate, reduce anxiety, and sustain motivation over time. For example, human-computer conversational chatbots can foster L2 learners’ willingness to communicate (Fathi et al., 2024); asynchronous speaking platforms can lower the pressure of real-time performance and thus mitigate speaking anxiety (Barkanyi and Brash, 2025); and online one-to-one conversation lessons that provide corrective feedback have been shown to improve speaking performance while at the same time creating a psychologically safer space for learners who feel anxious in face-to-face interaction (Tabuchi et al., 2024). By deliberately choosing oral-practice tools with these affect-supportive functions, teachers can build an online learning climate that is in some cases more inclusive than traditional classroom settings.

On the other hand, the findings also suggest that the design of ASR-supported speaking platforms should move beyond technical usability alone. In the present study, the GCLP appears to support affective variables to some extent through specific feedback and low-stakes practice, yet its design remains comparatively limited in social presence and interactional richness. This may help explain why students could perceive the platform as useful without those perceptions becoming especially strong correlates of speaking performance. Future development of GCLP-like platforms may therefore benefit from integrating features that not only maintain usability, but also strengthen affective support more directly, such as more varied speaking scenarios, richer interactional feedback, and opportunities for guided social participation. In this sense, the present findings suggest that the value of speaking technology may depend less on learners simply perceiving it as useful or easy to use, and more on whether its design meaningfully supports affective readiness for oral communication (Zhang et al., 2025; Li et al., 2025; Ren and Tan, 2025).

### 5.3 Limitation and future suggestions

Although this study provides valuable insights, several limitations should be acknowledged, and corresponding suggestions are provided to address each of the limitations. One limitation concerns the sample diversity and generalizability of this study. Although the 113 participants represented 34 L1 backgrounds across four continents, the cohort was homogeneous in two respects that matter for the present research question: (a) socio-academic setting—all were degree students enrolled in the same university programme; and (b) L2 proficiency—all were beginners preparing for HSKK3. Future studies should employ random sampling across multiple institutions, include non-degree programmes and heritage learners, and sample intermediate/advanced CSL learners. Collecting pre-specified covariates (e.g., prior L2 speaking hours, length of study, instructional context) will enable moderator and sensitivity analyses and improve external validity (Etikan et al., 2016).

Another limitation involves the instruments. HSKK captures global accuracy and fluency but omits interactive pragmatics; paired tasks or role-plays could be added to obtain a fuller picture of communicative competence in future studies. Similarly, affective and technological factors were measured using self-reported questionnaires. While self-report questionnaires are an efficient way to collect large-scale data, future studies could use alternative data sources to triangulate self-report data. For example, video would serve as a multi-source cross-check rather than a fully objective metric. Additionally, the total number of questionnaire items may have introduced response fatigue, particularly in the later sections of the survey, which could have affected response consistency.

Finally, the present study represents a small-scale teaching experiment; its sample and single-shot design necessarily limit the complexity of the relationships that can be modelled. Reverse or reciprocal relationships are also plausible; for example, learners with stronger speaking ability may be more likely to report more positive affective and platform perceptions. Also, the design is cross-sectional and the findings are correlational. Although participants were drawn from the same HSKK3 preparation track and were therefore broadly comparable in proficiency, baseline speaking proficiency was not included as a covariate in the main regression models. A logical next step is to extend the intervention to a full semester, recruit a substantially larger and more diverse cohort, and collect longitudinal platform logs (actual usage behaviour) together with staged measurements of behavioural intention. With these richer data, future research will be able to test a full structural-equation model that includes both the direct and indirect pathways through which technological and affective factors jointly shape CSL speaking development.

## 6 Conclusion

This study mapped associations between affective and technological factors and learners’ Chinese speaking proficiency in an ASR-supported platform, and compared the incremental explanatory power of these two sets of variables. The affective block—willingness to communicate, motivation, self-esteem, and anxiety—jointly accounted for over half of the variance in HSKK scores, whereas perceived usefulness and perceived ease of use provided only a modest incremental contribution. These results do not contradict documented benefits of technology for oral proficiency; rather, they suggest a boundary condition: under short ASR practice, affective readiness may be more consequential than baseline technology beliefs for explaining performance differences.

Because the design is cross-sectional and the analyses are correlational, the direction of influence cannot be established—stronger speaking ability may also foster more positive affect and technology perceptions. The practical implication is therefore two-fold: instructors and developers should continue to leverage ASR and related tools, while also prioritising affect-supportive design and pedagogy (e.g., features and practices that bolster WTC, motivation, and self-esteem and attenuate speaking anxiety). Future research should recruit larger, more diverse cohorts, adopt longitudinal or experimental designs, and test full mediation pathways to disentangle how affect and technology perceptions jointly drive growth in CSL speaking proficiency.

## Data Availability

The raw data supporting the conclusions of this article will be made available by the authors upon reasonable request, subject to ethical and privacy restrictions.
